# Cost effectiveness of epidural steroid injections to manage chronic lower back pain

**DOI:** 10.1186/1471-2253-12-26

**Published:** 2012-09-27

**Authors:** David K Whynes, Robert A McCahon, Andrew Ravenscroft, Jonathan Hardman

**Affiliations:** 1 School of Economics, University of Nottingham, Nottingham, NG7 2RD, UK; 2 Department of Anaesthesia, Nottingham University Hospitals NHS Trust, Queen’s Medical Centre, Nottingham, NG7 2UH, UK; 3 Department of Anaesthesia, Nottingham University Hospitals NHS, Nottingham City Hospital, Nottingham, NG5 1PB, UK; 4 Division of Anaesthesia and Intensive Care, University of Nottingham, Queen’s Medical Centre, Nottingham, NG7 2UH, UK

## Abstract

**Background:**

The efficacy of epidural steroid injections in the management of chronic low back pain is disputed, yet the technique remains popular amongst physicians and patients alike. This study assesses the cost effectiveness of injections administered in a routine outpatient setting in England.

**Methods:**

Patients attending the Nottingham University Hospitals’ Pain Clinic received two injections of methylprednisolone plus levobupivacaine at different dosages, separated by at least 12 weeks. Prior to each injection, and every week thereafter for 12 weeks, participants completed the EQ-5D health-related quality of life instrument. For each patient for each injection, total health state utility gain relative to baseline was calculated. The cost of the procedure was modelled from observed clinical practice. Cost effectiveness was calculated as procedure cost relative to utility gain.

**Results:**

39 patients provided records. Over a 13-week period commencing with injection, mean quality adjusted life year (QALY) gains per patient for the two dosages were 0.028 (SD 0.063) and 0.021 (SD 0.057). The difference in QALYs gained by dosage was insignificant (paired t-test, CIs -0.019 – 0.033). Based on modelled resource use and data from other studies, the mean cost of an injection was estimated at £219 (SD 83). The cost utility ratio of the two injections amounted to £8,975 per QALY gained (CIs 5,480 – 22,915). However, at costs equivalent to the tariff price typically paid to providers by health care purchasers, the ratio increased to £27,459 (CIs 16,779 – 70,091).

**Conclusions:**

When provided in an outpatient setting, epidural steroid injections are a short term, but nevertheless cost effective, means of managing chronic low back pain. However, designation of the procedure as a day case requires the National Health Service to reimburse providers at a price which pushes the procedure to the margin of cost effectiveness.

**Trial registration:**

ISRCTN 43299460

## Background

Chronic lower back pain has been managed by epidural steroid injection (ESI) for decades yet the use of the technique is contentious. On the one hand, recent reviews of clinical evidence published under the auspices of authoritative bodies such as the American Pain Society [1], the Cochrane Collaboration [2] and the UK’s Royal College of General Practitioners [3] have concluded that the effectiveness of ESIs remains unproven. Associated clinical guidelines, such as those of the UK’s National Institute for Health and Clinical Excellence [4], explicitly discourage the use of ESIs for non-specific low back pain. On the other hand, many pain management practitioners are robustly critical of such judgments [5,6]. Some reviewers have interpreted the research findings more positively [7,8], especially with respect to ESI’s apparent capacity to produce short-term benefits [9-11]. In part, judgments are conditioned by disputes over how the evidence should be interpreted [12,13].

Even as the scientific controversy over ESIs continues, the use of the technique in clinical practice proliferates. In Canada, for example, ESI is the most common pain procedure amongst anaesthesiologists [14]. In England’s National Health Service, the annual number of ESI procedures undertaken increased by 45 per cent between 2000 and 2010 [15], whilst the proportion of the USA Medicare population receiving an ESI more than doubled between 1997 and 2006 [16]. These trends reflect, presumably, the rising incidence of reported low back pain over time [17,18] and the likelihood that ESIs are deemed, at least by a sufficient number of practitioners and patients, to offer benefits.

Given that pain clinics are delivering ESIs for chronic low back pain in the face of disputed evidence, it would seem important to establish whether there are grounds for believing that ESIs offer value for money. In this paper, we assess the cost effectiveness of ESI. Using data obtained during a clinical trial, this cohort study compares costs in relation to effects for the same patients, based on a before-and-after evaluative design. Baseline data are compared with post-treatment data over a 12-week follow up period.

## Method

The Nottingham University Hospitals’ Pain Clinic hosted a prospective trial of the care of patients with chronic lower back pain, who were attending for routine repeat ESI. The principal clinical objective of the study was to investigate dose–response, by testing the hypothesis that an injection of methylprednisolone (MPA) 40 mg plus 25 mg levobupivacaine was less effective in ameliorating short-term (< 3 months) pain-related disability than one of MPA 80 mg plus 25 mg levobupivacaine. The primary clinical outcome was the observed change in the patients’ Oswestry Low Back Pain Disability Index (ODI) scores, the ODI being a widely-accepted condition-specific measure used in the management of spinal disorders [19].

Over a six month period, patients were invited to participate if they had been scheduled for an ESI at the Clinic, if they had received 2 or more ESIs in the previous 12 months, if they were currently experiencing pain and if their ODI score indicated moderate disability or worse. The study was conducted as a double-blinded crossover trial, with each participant receiving one 40 mg and one 80 mg MPA epidural injection in random order, separated by at least 12 weeks. Immediately prior to each injection, study participants completed an array of baseline questionnaires. Patients were then provided with further multiple copies of the questionnaires and were instructed to complete the same questionnaire array every week over the 12 weeks following each injection. They were permitted to continue all concurrent analgesic medications and received weekly telephone reminders to complete their questionnaires. Patient exited the study 12 weeks after their second ESI. In principle, therefore, each participant could complete 26 questionnaire arrays, in the form of two sequences of 13. Other than dose variation and data-gathering, patients were managed according to the Clinic’s normal practices.

The study was approved by the Institutional Review Board, Research Ethics Committee, and the Medicines & Healthcare Regulatory Authority (MHRA) UK prior to commencement, and was registered prior to the recruitment of the first participant (ISRCTN 43299460). Members of the study team obtained written informed consent from all study participants prior to randomization in accordance with the ethical principles that have their origin in the Declaration of Helsinki and the ICH (International Conference on Harmonisation) Guideline for Good Clinical Practice. Full details of the trial’s organization and its clinical findings have been published elsewhere [20].

Alongside the ODI, the questionnaire array included the EQ-5D health-related quality of life instrument [21]. This is a well-validated instrument, widely used for constructing the health state utilities which inform cost utility analysis [22]. The EQ-5D questionnaire comprises two components. First, the subject classifies his or her health state by indicating the degree of severity of problem experienced in each of five independent domains. Any health state so classified can be converted to a single utility weight or “index score”, using a set of values derived from a sample of the general population. Second, the subject indicates his or her current state on a visual analogue scale (VAS), within the range “worst imaginable” to “best imaginable” health state. As the EQ-5D is a quality of life measure, higher scores imply higher quality. The nominal range of the EQ-5D index score is 0–1, but negative scores are possible for health states deemed to be worse than death. The VAS range is 0–100.

Index scores were calculated from subjects’ EQ-5D classifications using the UK tariff [23]. The patient’s utility gain or loss consequent upon an injection was expressed in quality-adjusted life years (QALYs). Longitudinal health state utility gain can be represented as the area under the curve of index scores against time, relative to the pre-treatment baseline score [24]. We therefore calculated the QALY gain for each patient following each of the 40 mg and the 80 mg injections as the sum of the differences between each weekly index score and the patient’s baseline index score over the 12 week follow-up, divided by 52. A degree of incomplete adherence to questionnaire completion was anticipated and, where an index score was missing for a particular week in a sequence, it was interpolated from adjacent values. Thereafter, we calculated the mean QALY gains from ESI for each dosage.

Our cost model considered only the resource use of the health care provider. To calculate the cost of an ESI in the Pain Clinic, we modeled resource use and translated this into monetary values using unit resource costs. Cost estimates published in previous studies provided both inputs to our model and confidence intervals. All costs were expressed in £UK at 2010 prices. All confidence intervals (CIs) reported subsequently are at 95 per cent. The incremental cost effectiveness ratio (ICER) was calculated as the estimated cost of the procedure relative to utility gains. As unit costs and utilities were uncorrelated, the estimates of CIs for the ICER were obtained via Fieller’s method using a proprietary calculator [25].

## Results

### Outcomes

**Figure 1:**
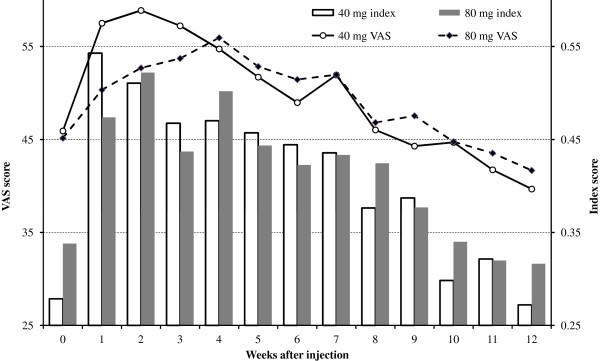
Mean EQ-5D scores.

Of the 41 patients originally recruited, 37 returned EQ-5D sequences for both dosages, and a further two supplied a sequence for one dosage only. Eleven patients returned sequences containing one or more missing observations, thereby necessitating interpolation. The interpolation of a single index score was necessary for eight patients, two scores for another patient and three scores for a further two. Figure 1 portrays the mean index and VAS scores for the patients throughout the sequences. It is evident that, following injection of either 40 mg or 80 mg, health-related quality of life improved rapidly from baseline (week 0). By week 2, the mean EQ-5D index score was significantly higher than that at baseline for both dosages. When patients received the 40 mg and the 80 mg ESIs, the increases in mean index scores were 0.23 (CIs 0.09 – 0.37) and 0.18 (CIs 0.04 – 0.32), respectively. By week 12, however, index scores had reverted to baseline: differences between week 0 and week 12 index scores were insignificant, at 0.01 (CIs -0.14 – 0.16) and 0.02 (CIs -0.14 – 0.18), respectively. Each of the 13 week-for-week comparisons of mean index and VAS scores between the 40 mg and 80 mg dosages revealed an insignificant difference (t-test, all p = 0.20 or greater). Essentially, the EQ-5D results corroborated the inferences which had been made using the ODI data [20]. The ODI had also detected both improvements in average short-term health status following ESI under either dosage and an absence of significant differences between the week-by-week changes across the two dosages.

The total QALYs gained over baseline by the patients over each of the 13-week measurement sequences was, on average, 0.028 (SD 0.063) when receiving the 40 mg dose injection and 0.021 (SD 0.057) when receiving the 80 mg dose injection. The difference in QALYs gained by dosage was insignificant (paired t-test, CIs -0.019 – 0.033). As each patient received one injection at each dose, the QALYs gained over two treatment episodes was the sum of the gains from each of the doses. This amounted to, on average, 0.049 QALYs (SD 0.093).

### Costs

The ESI procedure at the Clinic involved an anaesthesiologist (consultant grade) working with two registered nurses and two care assistants. The procedure was conducted without recourse to fluoroscopic guidance. Labour costs were derived from national estimates which included salaries, insurance and pension contributions, labour-related hospital overheads and capital development costs [26]. For the above three grades, these costs were £169, £47 and £24, respectively, per patient-contact-hour. Eight patients were routinely scheduled for a half-day session and we therefore assumed an average of 30 minutes treatment time per patient. As a result, total labour costs amounted to £156 per ESI.

The cost of disposables used during the procedure, such as drugs, dressings, gown and cannula, was derived from an earlier detailed audit of ESIs in the management of sciatica [27]. As the principal drug employed in that earlier study differed from that used in this one (MPA), we substituted the cost of the latter for that of the former, resulting in a total cost of disposables of £27. The cost of MPA itself was relatively low (around £5) and, owing to the standardisation of vial capacity, did not vary by the size of dose used. Finally, we assigned a mark-up of 20% on variable costs to cover overheads such as the use of hospital facilities and utilities. This proportion follows that used in the sciatica evaluation but also corresponds closely to the proportion of expenditure devoted to areas other than personnel, clinical services and supplies in our own hospital [28]. Combining these components, our model yielded a cost of £219 per ESI delivered.

Modelled costs provide only a single value. To introduce dispersion around this estimate we searched the literature for previous cost estimates. The sciatica study, above, was one of only six which we were able to locate, in which ESI costs had been presented explicitly. Table 1 provides details of the six studies, including country and year of cost estimation. In three cases (labeled “tariff”), the costs of an ESI were those allowable under the country’s national health insurance scheme. In the remaining cases, costs resulted from audits of the ESI procedure (a micro-costing or “bottom-up” approach). We used the Institute of Education cost converter [29] to take account of inflation and exchange rates. Our own cost estimate lay within 4 per cent of the mean of the six previous values. Adding our model result to the list of estimates, we calculated a standard deviation of 83 for the seven observations and used this as our dispersion parameter for unit cost.

**Table 1:** Cost estimates

**Source**	**Country**	**Date**	**Type**	**Cost**
				**(£UK, 2010cp**
[30]	Belgium	1999	Tariff	299
[31]	Australia	1999	Tariff	196
[32]	UK	1999	Audit	94
[27]	UK	2002	Audit	189
[33]	Netherlands	2005	Audit	153
[34]	USA	2007	Tariff	335
Mean of the above				211

Under its present organizational form, the English National Health Service (NHS) is divided into care providers, such as hospitals and clinics, and care purchasers, who hold publicly-supplied funds. Providers are recompensed by purchasers for services delivered, on the basis of national tariffs assigned to defined or coded procedures. The tariff, in other words, is that which the NHS is obliged to pay for the service, as opposed to that which the service might cost. That having been said, tariffs are set from reference costs, which are themselves estimated from the financial returns of individual providers who are required to employ a standardized accounting frame. Reference costs depend on the service definition and context. Most providers in England have classified an ESI for chronic pain as a “major pain procedure”, coded AB04Z. Undertaking the procedure as a “day case” entails formally admitting the patient to the hospital, treating and discharging within the same day. The mean reference cost of a day case AB04Z was £670 (SD 248), subject to local variations [35]. However, the procedure can also be delivered on an outpatient basis, whereby the patient attends for treatment but is not admitted. The mean reference cost for outpatient AB04Z was £145 (SD 101). Nearly 89,000 AB04Z procedures were performed in English hospitals in 2010–11, around 94 per cent of which were day cases.

### Cost-utility

From the above, the average patient receiving two ESIs at different dosages incurred modeled costs of £438 (SD 166) for an expected gain of 0.049 QALYs (SD 0.093). When combined, these estimates for outcome and cost produced an ICER of £8,975 per QALY gained (CIs 5,480 – 22,915) for an “average” ESI plus analgesics compared with analgesics alone. This placed the intervention below the current threshold for cost effectiveness in England, namely, the range £20-30,000 per QALY gained [36]. Replacing the modeled cost estimate with the reference cost for delivery in an outpatient setting caused the ICER to fall £5,943 (CIs 3,462 – 15,338), thereby making the intervention appear even more cost effective. Replacing the modeled cost estimate with the reference cost for ESI as a day case caused the ICER to rise to £27,459 (CIs 16,779 – 70,091). This mean value is at the upper end of the acceptable range for cost effectiveness in the English NHS.

## Discussion

As assessed using the EQ-5D instrument, patients evidently followed an “inverted-U-shaped” quality of life path over time (Figure 1). This is consistent with the paths reported in previous observational studies, although these studies used pain-specific outcome measures and recorded less frequently [37,38]. The effect was reported to be greatest up to 4 weeks following injection, with progressive reduction in impact in the months following. Similar findings have been reported in a dosage trial [39].

Comparisons between our estimates and those from two ESI pain trials are instructive. The PINE trial in the Netherlands [40] included around 600 patients with acute herpes zoster. As with our own study, it compared analgesics only with analgesics plus ESI using MPA. Even though it measured short term EQ-5D outcomes less frequently, at 4 weeks and at 12 weeks only, the reported trend in outcome was similar. Compared with analgesics only, ESI improved index scores significantly at 4, but not at 12, weeks. However, at an estimated 0.01 QALYs gained per patient [33], the net health gain was smaller than for our subjects. By way of explanation for the difference, the PINE measurement schedule was less intensive. More widely spaced readings imply less precision and would result in undervaluation if, as our study has concluded, noticeable benefits begin to accrue almost immediately after injection. Also, the PINE patients’ conditions were acute rather than chronic and their baseline EQ-5D-measured quality of life was higher than that of our patients. Their pain was less severe, and it would seem probable that the marginal benefit of ESI is lower when pre-intervention quality of life is higher.

An English trial of multiple ESIs for sciatica included around 230 patients [27]. As with the PINE study, these were assessed twice only in the short term, at 3 and at 6 weeks. Against a placebo saline injection, the estimated health gain was even smaller, at 0.006 QALYs per patient, and almost all this accrued at 3 weeks. Again by way of explanation, quality of life outcomes for the sciatica trial were measured using the SF36 instrument, translated to the SF-6D and its associated utilities. There is robust evidence from parallel instrumentation studies that, for a given health improvement, the utility gain when measured using the SF-6D is significantly smaller than when measured by the EQ-5D [41]. Also, and unlike our patients, the sciatica patients were a mixture of chronic and acute, and the health gain amongst the acute patients was smaller. Finally, the sciatica trial was not so much a trial of the procedure as of the active ingredient, because all subjects received an injection.

A common feature of both of these previous trials was that the subjects had no record of successful treatment by ESI. Indeed, previous receipt of an ESI for pain was an explicit exclusion criterion for the sciatica trial. In contrast, all our patients had received ESIs at the Pain Clinic on previous occasions. One of the few areas of agreement in the debate over the efficacy of ESIs is that not all people experiencing chronic back pain respond to treatment. It therefore follows that differential bias in recruitment must also contribute to explaining the differences in findings. Trials requiring subjects to have no experience of ESI in pain relief are more likely to recruit people for whom the technique will prove ineffectual, thereby lowering the average health gain recorded. In contrast, the clientele of any clinic offering routine care will be dominated by patients for whom the treatment is efficacious. Many of the patients unresponsive to ESI, therefore, would have discontinued treatment prior to being recruited into our study.

Combining all this information, there appear good grounds for suspecting the presence of a placebo effect within ESI’s overall treatment effect. Patient-reported outcomes in both the PINE study [33] and our own indicated that ESI produced significant patient-reported short-term health gains, on average. However, both the sciatica study [27] and our own suggested that the marginal impact of the active ingredient in the injection might have been small. The sciatica study detected only modest gains from an active injection compared with a saline placebo, and others have reported similarly [42]. We identified no significant differences in utilities following ESIs with different dosages. Identifying the magnitude of the placebo effect itself could prove difficult. It would require a trial with two randomisations rather than one, namely, between injection and “no treatment” and between active and placebo ingredients. In fact, such a design has already been rejected in the studies cited, although with different objections. Whilst the sciatica investigators deemed it unethical to include a “no treatment” arm, the PINE investigators considered a placebo arm to be unethical. Ethical considerations aside, such a trial might fail to recruit, on the expectation that few patients would risk randomisation away from a well-established (if not well-proven) treatment.

This study has demonstrated cost effective quality of life gains from two ESI episodes over a six month period. How this practice might fit into a longer term management scenario remains unclear, both from this study and from others. In the absence of further injections, for example, patients’ symptoms could eventually stabilise at or above the pre-injection baseline or, alternatively, deteriorate to poorer states of health. Both results have been reported [43,44]. Were injections to be repeated regularly over the longer term, it is unclear whether each subsequent ESI would produce the same effect as its predecessor or whether diminishing returns would prevail. It is probable that a belief in a repeated benefit influences contemporary practice; a survey of US anaesthesiologists revealed that the average maximal number of ESIs per patient was in excess of four per year [45]. In spite of the popularity of serial injection, however, a review found only limited, and contradictory, evidence for offering patients repeated ESIs [46].

We noted that our results permitted different interpretations of the ICER, depending on whether costs were modelled from estimates of resource use or assessed at tariff values. Compared with the ICER using the modelled costs, the ICER using day case reference costs was around 3.4-times higher, and the ICER using outpatient reference costs was around two-thirds lower. The two other studies reported a similar phenomenon but with even more extreme disparities. The protocol of the English sciatica study allowed for multiple injections to be carried out [27]. The NHS tariff cost of the regimen specified by the protocol was 7.9-times greater than the costs estimated from measured resource use, with corresponding implications for the ICER. In the PINE study, the ICER based on the tariffs for ESIs allowed by the Netherlands insurance companies’ reimbursement scheme was 4.6-times higher than that estimated using the observed costs of the procedures [33]. The authors concluded that the balance of cost effectiveness tipped as the divergence between tariff and resource cost increased, and the conclusions from our own study are essentially equivalent.

How a procedure is classified for reimbursement is more a matter of accounting than of clinical practice. In this context, the Audit Commission, which monitors efficiency in public spending, has recently commented on the inconsistent classification of short-term treatments throughout the NHS [47]. It concludes that discrepancies between hospitals arise less from lack of clarity in guidance (although this is certainly present) and more from financial incentives. As outpatient treatment is usually recompensed at a lower tariff than day case treatment, many providers choose to provide essentially the same service and designate it as the latter rather than as the former. The Audit Commission notes that the NHS is therefore paying far more for services than might otherwise be necessary and that inaccuracies are being introduced into procedures’ estimated reference costs. We would add that, according to our ESI study, the designated setting of service delivery could prove significant in determining whether or not a procedure could be considered unequivocally cost effective.

Cost of illness studies [48] generally demonstrate that the direct costs of treating back pain are less than the indirect costs of the condition. These include both absences from work resulting in lost production and decreased productivity on the part of those who continue working despite being in pain. As we measured only health gains and the direct costs of treatment in this study, the contribution of ESIs to reducing the non-NHS costs of back pain has not been accounted for. However, we would suppose that pain reduction effected by an ESI would, at worst, have no effect on absence from work and productivity losses, and it might well reduce them. To complete the ICER calculation from a social perspective, therefore, the direct costs of treatment would have to be partially offset by reductions in the indirect costs of pain. By implication, the ICERs we have estimated represent maximum values. Evaluated from a social perspective, the ICERs would be lower than those presented earlier, i.e. the procedure would be judged more, rather than less, cost effective.

## Conclusions

Although the efficacy of epidural steroid injections is the subject of debate, the technique is widely-used. Our study indicates short term effectiveness in improving health-related quality of life. Our results suggest that steroid injections can be a cost effective means of managing chronic low back pain. However, designation of the procedure as a day case is likely to require the National Health Service to reimburse providers at a price which pushes the procedure to the margin of cost effectiveness.

## Competing interests

The authors declare that they have no competing interests.

## Authors’ contributions

RAM and JH designed the study. AR prepared the drugs and treated the patients. RAM and AR collected the data. DKW analysed the data and drafted the paper. All authors read and approved the final manuscript.

## Pre-publication history

The pre-publication history for this paper can be accessed here:

http://www.biomedcentral.com/1471-2253/12/26/prepub

## References

[B1] Chou R Loeser J Owens DK Rosenquist RW Atlas SJ Baisden J Carragee EJ Grabois M Murphy DR Resnick DK Interventional therapies, surgery, and interdisciplinary rehabilitation for low back pain: an evidence-based clinical practice guideline from the American Pain Society Spine 2009 34 10 1066 1077 10.1097/BRS.0b013e3181a1390d 19363457

[B2] Staal JB de Bie RA de Vet HCW Hildebrandt J Nelemans P Injection therapy for subacute and chronic low back pain: an updated Cochrane review Spine 2009 34 1 49 59 10.1097/BRS.0b013e3181909558 19127161

[B3] Savigny P Kuntze S Watson P Underwood M Ritchie G Cotterell M Hill D Browne N Buchanan E Coffey P Low back pain: early management of persistent non-specific low back pain (full guideline) 2009 National Collaborating Centre for Primary Care and Royal College of General Practitioners, London

[B4] National Institute for Health and Clinical Excellence Low back pain: early management of persistent non-specific low back pain (clinical guideline 88) 2009 NIHCE, London

[B5] Kmietowicz Z President of British Pain Society is forced from office over NICE guidance on low back pain Br Med J 2009 339 b3049 10.1136/bmj.b3049

[B6] Manchikanti L Datta S Gupta S Munglani R Bryce DA Ward SP Benyamin RM Sharma ML Helm S Fellows B A critical review of the American Pain Society clinical practice guidelines for interventional techniques: part 2 - therapeutic interventions Pain Physician 2010 13 E215 E264 20648212

[B7] Deer T Ranson M Kapural L Diwan SA Guidelines for the proper use of epidural steroid injections for the chronic pain patient Techniques in Regional Anesthesia and Pain Management 2009 13 288 295 10.1053/j.trap.2009.06.010

[B8] Levin JH Prospective, double-blind, randomized placebo-controlled trials in interventional spine: what the highest quality literature tells us Spine J 2009 9 690 703 10.1016/j.spinee.2008.06.447 18789773

[B9] Weinstein SM Herring SA Lumbar epidural steroid injections Spine J 2003 3 37S 44S 10.1016/S1529-9430(02)00560-0 14589216

[B10] Armon C Argoff CE Samuels J Backonja M-M Use of epidural steroid injections to treat radicular lumbosacral pain: report of the Therapeutics and Technology Assessment Subcommittee of the American Academy of Neurology Neurology 2007 68 723 729 10.1212/01.wnl.0000256734.34238.e7 17339579

[B11] Cohen SP White RL Kurihara C Larkin TM Chang A Griffith SR Gilligan C Larkin R Morlando B Pasquina PF Epidural steroids, etanercept, or saline in subacute sciatica: a multicenter, randomized trial Ann Intern Med 2012 156 551 559 22508732 10.7326/0003-4819-156-8-201204170-00397

[B12] Hopayian K Mugford M Conflicting conclusions from two systematic reviews of epidural steroid injections for sciatica: which evidence should general practitioners heed ? Br J Gen Pract 1999 49 57 61 10622020 PMC1313321

[B13] Chou R Same trials, different conclusions: sorting out discrepancies between reviews on interventional procedures of the spine Spine J 2009 9 679 689 10.1016/j.spinee.2009.05.003 19540814

[B14] Peng PWH Castano ED Survey of chronic pain practice by anesthesiologists in Canada Can J Anaesth 2005 52 4 383 389 10.1007/BF03016281 15814753

[B15] NHS Information Centre for Health and Social Care Main procedures and interventions: 3 character http://www.hesonline.nhs.uk/Ease/servlet/ContentServer?siteID=1937&categoryID=205

[B16] Manchikanti L Pampati V Boswell MV Smith HS Hirsch JA Analysis of the growth of epidural injections and costs in the Medicare population: a comparative evaluation of 1997, 2002, and 2006 data Pain Physician 2010 13 199 212 20495584

[B17] Freburger JK Holmes GM Agans RP Jackman AM Darter JD Wallace AS Castel LD Kalsbeek WD Carey TS The rising prevalence of chronic low back pain Arch Intern Med 2009 169 3 251 258 10.1001/archinternmed.2008.543 19204216 PMC4339077

[B18] Harkness EF Macfarlane GJ Silman AJ McBeth J Is musculoskeletal pain more common now than 40 years ago ?: two population-based cross-sectional studies Rheumatology 2005 44 890 895 10.1093/rheumatology/keh599 15784630

[B19] Fairbank JCT Pynsent PB The Oswestry Disability Index Spine 2000 25 22 2940 2953 10.1097/00007632-200011150-00017 11074683

[B20] McCahon RA Ravenscroft A Hodgkinson V Eveley R Hardman J A pilot study of the dose–response of caudal methylprednisolone with levobupivacaine in chronic lower back pain Anaesthesia 2011 66 7 595 603 10.1111/j.1365-2044.2011.06764.x 21564047

[B21] Brooks R, Rabin R, Charro F The measurement and valuation of health status using EQ-5D: a European perspective 2003 Kluwer Academic Publishers, Dordrecht

[B22] Brazier J Valuing health states for use in cost-effectiveness analysis PharmacoEconomics 2008 26 9 769 779 10.2165/00019053-200826090-00007 18767897

[B23] Dolan P Modeling valuations for EuroQol health states Med Care 1997 35 11 1095 1108 10.1097/00005650-199711000-00002 9366889

[B24] Gold MR, Siegel JE, Russell LB, Weinstein MC Cost-effectiveness in health and medicine 1996 Oxford University Press, New York

[B25] GraphPad Software Inc 2012 , La Jolla, California, USA http://www.graphpad.com/QuickCalcs/ErrorProp1.cfm

[B26] Curtis L Unit costs of health and social care 2010 2010 Personal Social Services Research Unit, University of Kent, Canterbury

[B27] Price C Arden N Coglan L Rogers P Cost-effectiveness and safety of epidural steroids in the management of sciatica Health Technol Assess 2005 9 33 1 58 16095548 10.3310/hta9330

[B28] NUH NHS Trust Annual Report 2009/10 2010 Nottingham University Hospitals NHS Trust, Nottingham

[B29] Campbell & Cochrane Economics Methods Group and the Evidence for Policy and Practice Information and Co-ordinating Centre CCEMG – EPPI-Centre Cost Converter (v.1.2) http://eppi.ioe.ac.uk/costconversion/default.aspx

[B30] van Zundert J van Kleef M Low back pain: from algorithm to cost-effectiveness ? Pain Pract 2005 5 3 179 189 10.1111/j.1533-2500.2005.05303.x 17147580

[B31] Jackson C Broadhurst N Bogduk N An audit of the use of epidural injections for back pain and sciatica Aust Health Rev 2003 26 1 34 42 10.1071/AH030034 15485372

[B32] McGregor AH Anjarwalla NK Stambach T Does the method of injection alter the outcome of epidural injections ? J Spinal Disord 2001 14 6 507 510 10.1097/00002517-200112000-00008 11723401

[B33] Opstelten W van Wijck AJ van Essen GA Moons KG Verheij TJ Kalkman CJ van Hout BA Cost effectiveness of epidural injection of steroids and local anesthetics for relief of zoster-associated pain Anesthesiology 2007 107 678 679 10.1097/01.anes.0000282005.39025.ed 17893478

[B34] Office of Inspector General Inappropriate Medicare payments for transformaminal epidural injection services (OEI-05-09-00030) 2010 Department of Health and Human Services, Washington

[B35] Department of Health NHS Payment by Results 2010-11 National Tariff Informationhttp://data.gov.uk/dataset/payment-by-results-2010-11-national-tariff-information

[B36] National Institute for Health and Clinical Excellence Social value judgments: principles for the development of NICE guidance 2008 2 NICE, London 27905706

[B37] Loy TTT Epidural steroid injection for sciatica: an analysis of 526 consecutive cases with measurements and the whistle test J Orthop Surg 2000 8 1 39 44 10.1177/230949900000800108 12468874

[B38] Delport EG Cucuzzella AR Marley JK Pruitt CM Fisher JR Treatment of lumbar spinal stenosis with epidural steroid injections: a retrospective outcome study Arch Phys Med Rehabil 2004 85 3 479 484 10.1016/S0003-9993(03)00472-6 15031837

[B39] Owlia MB Salimzadeh A Alishiri G Haghighi A Comparison of two doses of corticosteroid in epidural steroid injection for lumbar radicular pain Singapore Med J 2007 48 3 241 245 17342295

[B40] van Wijck AJM Opstelten W Moons KGM van Essen GA Stolker RJ Kalkman CJ Verheij TJM The PINE study of epidural steroids and local anaesthetics to prevent postherpetic neuralgia: a randomised controlled trial Lancet 2006 367 219 224 10.1016/S0140-6736(06)68032-X 16427490

[B41] Sach TH Barton GR Jenkinson C Doherty M Avery AJ Muir KR Comparing cost-utility estimates: does the choice of EQ-5D or SF-6D matter? Med Care 2009 47 8 889 894 10.1097/MLR.0b013e3181a39428 19584759

[B42] Iversen T Solberg TK Romner B Wilsgaard T Twisk J Anke A Nygaard Ø Hasvold T Ingebrigtsen T Effect of caudal epidural steroid or saline injection in chronic lumbar radiculopathy: multicentre, blinded, randomised controlled trial Br Med J 2011 343 d5278 10.1136/bmj.d5278 21914755 PMC3172149

[B43] Karppinen J Malmivaara A Kurunlahti M Kyllönen E Pienimäki T Nieminen P Ohinmaa A Tervonen O Vanharanta H Periradicular infiltration for sciatica: a randomized controlled trial Spine 2001 26 9 1059 1067 10.1097/00007632-200105010-00015 11337625

[B44] Wilson-MacDonald J Burt G Griffin D Glynn C Epidural steroid injection for nerve root compression: a randomised controlled trial J Bone Joint Surg Am 2005 87-B 3 352 355 10.1302/0301-620X.87B3.15338 15773645

[B45] Cluff R Mehio AK Cohen SP Chang Y Sang CN Stojanovic MP The technical aspects of epidural steroid injections: a national survey Anesth Analg 2002 95 2 403 408 12145061 10.1097/00000539-200208000-00031

[B46] Novak S Nemeth WC The basis for recommending repeating epidural steroid injections for radicular low back pain: a literature review Arch Phys Med Rehabil 2008 89 543 552 10.1016/j.apmr.2007.11.008 18295635

[B47] Commission A By definition: improving data definitions and their use by the NHS 2011 Audit Commission, London

[B48] Dagenais S Caro J Haldeman S A systematic review of low back pain cost of illness studies in the United States and internationally Spine J 2008 8 8 20 10.1016/j.spinee.2007.10.005 18164449

